# Evaluating Fit Indices for Multivariate *t*-Based Structural Equation Modeling with Data Contamination

**DOI:** 10.3389/fpsyg.2017.01286

**Published:** 2017-07-28

**Authors:** Mark H. C. Lai, Jiaqi Zhang

**Affiliations:** School of Education, University of Cincinnati Cincinnati, OH, United States

**Keywords:** structural equation modeling, robustness, outliers, data contamination, fit indices

## Abstract

In conventional structural equation modeling (SEM), with the presence of even a tiny amount of data contamination due to outliers or influential observations, normal-theory maximum likelihood (ML-Normal) is not efficient and can be severely biased. The multivariate-*t*-based SEM, which recently got implemented in Mplus as an approach for mixture modeling, represents a robust estimation alternative to downweigh the impact of outliers and influential observations. To our knowledge, the use of maximum likelihood estimation with a multivariate-*t* model (ML-*t*) to handle outliers has not been shown in SEM literature. In this paper we demonstrate the use of ML-*t* using the classic Holzinger and Swineford (1939) data set with a few observations modified as outliers or influential observations. A simulation study is then conducted to examine the performance of fit indices and information criteria under ML-Normal and ML-*t* in the presence of outliers. Results showed that whereas all fit indices got worse for ML-Normal with increasing amount of outliers and influential observations, their values were relatively stable with ML-*t*, and the use of information criteria was effective in selecting ML-normal without data contamination and selecting ML-*t* with data contamination, especially when the sample size was at least 200.

Although identification of outliers and influential observations is a standard practice in regression models, less attention has been given to such issues in structural equation modeling (SEM) (Pek and MacCallum, 2011). Nevertheless, as pointed out in previous literature (e.g., Yuan and Bentler, 2001; Yuan and Zhong, 2013), normal-theory based SEM is not robust to data contamination, and a small proportion of outliers and influential observations can bias parameter estimation, the likelihood ratio test statistic (LRT; also commonly referred to as the model χ^2^), and fit indices based on LRT. While robust modeling by replacing the normality assumption with one that assumes the error terms follow a heavier-tailed *t* distribution has long been discussed in regression models and multilevel models (e.g., Pinheiro et al., 2001; Gelman and Hill, 2006), not until recently are multivariate-*t* SEM models accessible to researchers, and there has been very little research on the usefulness of such models in the presence of data contamination. In this paper we first provide brief background information on the use of the multivariate *t* distribution for robust SEM modeling. We then demonstrate with a real data set how five outlying cases can have a severe impact on model fit and parameter estimates under normal-theory SEM (ML-Normal), and show that estimation using a *t* model (ML-*t*) produces similar inferences with and without data contamination. Finally, we conduct a simulation study to evaluate the performance of commonly used fit indices of ML-Normal, ML-*t*, and the use of Huber-type weights with and without data contamination, and the effectiveness of information criteria in selecting between ML-Normal and ML-*t*, across conditions of model misspecifications, sample sizes, and proportions of outliers and influential observations.

## Outliers and influential observations

Whereas topics related to outliers, or more generally *data contamination*, are commonly discussed in quantitative research methodology textbooks, in practice researchers do not always agree on their definitions and how best to handle them. For instance, in a review of organizational research, Aguinis et al. (2013) found 14 different definitions of outliers (which include but are not limited to cases with high leverage and with large influence on parameter estimates and model fit) and 20 different ways to handle them. Also, outliers of different nature require different treatments, and Aguinis et al. summarized the definitions of outliers in three categories: (a) those due to correctable errors such as input error, (b) those exhibiting idiosyncratic characteristics and of interest themselves (c) those exerting disproportionately large influence on the substantive conclusion regarding a model of interest. In this paper we focus on robust inference in SEM with data contamination in category (c).

Following Pek and MacCallum (2011), in this study we distinguish between *outliers* and *influential observations* for SEM, as they may have differential impacts on parameter estimation and model fit indices. Outliers are cases that lie far away from most other data points. In regression with only one response variable, outliers are cases with a large deviation from its predicted value based on the regression line. In multivariate analyses such as SEM, the distance of an observation from the center of most of the data points is commonly quantified by the Mahalanobis distance (*d*), where:

(1) $$d_{i}=\sqrt{{\left(y_{i}-\mu \right)}^{⊤}\Sigma (y_{i}-\mu )},$$

**y**_*i*_ = [*y*_1*i*_, …, *y*_*ki*_] is the data vector for the *i*th observation on *p* observed variables, **μ** and **Σ** are the mean vector and the covariance matrix of the *p* observed variables. On the other hand, influential observations are those that exert large influence on model fit and parameter estimation; in other words, parameter estimates and model fit indices will show relatively large changes when the influential cases are removed. Despite the conceptual differences between outliers and influential observations, they are not mutually exclusive as some outliers can also exert strong influence on the results.

Although in this paper we focus on methods to obtain fit indices and parameter estimates that are robust to extreme observations without necessarily identifying the outliers and influential observations, researchers are generally recommended to carefully inspect observations and identify correctable data entry errors and truly idiosyncratic observations. Examples of techniques for identifying outliers and influential observations in SEM were Cook's distance, Mahalanobis distance, and likelihood distance. Readers can consult Aguinis et al. (2013), Pek and MacCallum (2011), and Yuan and Zhang (2012) for more in-depth discussions on tools and procedures for identifying outliers and influential observations.

Here we borrow the notations from Asparouhov and Muthén (2015) for the linear SEM model and define outliers and influential observations as discussed in Yuan and Zhong (2008, 2013). For a model with *p* observed variables measuring *q* latent variables, we assume that the observed *p*-variate observed variable **Y** has a measurement model:

(2) Y=ν+Λη+e,

where **ν** is a *p* × 1 vector of measurement intercepts, **Λ** is a *p* × *q* factor loading matrix, and **e** is a *p* × 1 random vector containing measurement error terms. **η** is a *q*-variate latent variable with a structural model:

(3) η=α+Bη+ΓX+ξ,

where the effects among latent factors were captured by **B**, the effects of exogenous covariates **X** were captured by **Γ**, **ξ** is a random vector of disturbance terms, and **α** contains the latent regression intercepts. It is common to impose the normality assumption such that the joint distribution of **e** and **ξ** is multivariate normal, with:

(4) $$(e,\xi )\sim N_{p+q}\left(0,\left[\begin{array}{cc} \Theta & 0\\ 0 & \Psi \end{array}\right]\right).$$

As discussed in Yuan and Zhong (2008), outliers in SEM have large values of **e**, and will inflate the covariance matrix of the outcome variables **Σ**. However, it may or may not have large values in **η**. On the other hand, influential observations have extreme values in **ξ** and will inflate **Ψ** and also **Σ**. Influential observations can be good or bad: good influential observations have extreme **ξ** but not extreme **e** values, and will not negatively impact model fit as it is not considered outliers; bad influential observations, on the other hand, have both extreme **ξ** and **e** values, and will negatively impact model fit.

### Impact of outliers and influential observations

Under the normal model, a very small portion of outliers and influential observations can have a huge impact on parameter estimates and model fit. For example, Yuan and Bentler (2001) showed mathematically that existence of outliers can greatly inflate the Type I error rates of LRT and related test statistics adjusting for non-normality under ML-Normal, and the LRT statistic could be inflated by more than five times in values in an example given in Yuan and Zhong (2008); Yuan and Zhong (2008) also showed that in confirmatory factor analysis (CFA), about 3% of outliers could substantially bias the factor loading estimates by more than 50% and inflate the latent factor variance and covariance estimates by 3–10 times, whereas 3% of bad influential observations could produce even greater biases on all parameter estimates in CFA. Yuan and Zhong (2013) showed mathematically and illustrated with modified real data sets that outliers lead to worse fit indices such as RMSEA and CFI, whereas bad influential observations can lead to worse RMSEA but also *better* CFI in some situations. In summary, a few outliers and bad influential observations can lead to biased and inefficient parameter estimates and produced misleading and sometime contradictory information about model fit.

Despite the documented impact of outliers and influential observations, detection and diagnostics of such observations were rarely performed and reported in real research, and the use of SEM methods that are robust to data contamination has been scarce. For example, Aguinis et al. (2013) reviewed 232 methodological and substantive journal articles in organizational science journals that addressed issues about outliers and influential observations, and only five of them were related to SEM, despite the popularity of SEM in the past two decades. One possible reason is that practical guidelines on handling outliers and influential observations were developed more recently (e.g., Pek and MacCallum, 2011; Aguinis et al., 2013). Another possible reason is that existing methods for detecting and handling outliers and influential observations in SEM require researchers to use specialized programs (e.g., Sterba and Pek, 2012; Yuan and Zhang, 2012), thus creating additional burden for researchers if they are not familiar with those programs.

### Existing robust estimation methods in SEM

Because a small proportion of outliers and bad influential observations can produce invalid assessment of model fit and parameter estimates, it is important to have methods that produce consistent and efficient estimation and give robust model fit information in the presence of data contamination, assuming that those extreme values are not due to correctable errors (e.g., data entry errors) and the goal is to obtain inferences based on the majority of the sample. One such method in SEM is to replace the squared loss function in estimating the mean vector and covariance matrix by one that downweighs cases exerting unproportionally large influence on the model (Yuan and Bentler, 1998a,b, 2000; Yuan et al., 2000, 2004), which has been commonly used in robust regression. One popular choice of the weight function is the Huber-type weight function, which replaces the squared loss function by a linear function when the Mahalanobis distance of a case exceeds a prespecified cutoff, *u*, where *u*^2^ is the (1−φ)th quantile of a chi-square distribution. In other words, φ is the theoretical proportion of cases to be downweighed under normality with no data contamination.

In the two-stage robust method (TSR; Yuan and Bentler, 1998a), one first obtains robust means and covariances estimates with Huber-type weights:

$$\begin{array}{l} {\hat{\mu }}_{\text{TSR}}=\frac{{\sum^{\text{​}}}_{i=1}^{n}w_{1}(d_{i})y_{i}}{{\sum^{\text{​}}}_{i=1}^{n}w_{1}(d_{i})},\\ {\hat{\Sigma }}_{\text{TSR}}=\frac{1}{n}\overset{n}{\underset{i=1}{\sum^{\text{​}}}}w_{2}(d_{i})(y_{i}-{\hat{\mu }}_{\text{TSR}}){\left(y_{i}-{\hat{\mu }}_{\text{TSR}}\right)}^{⊤}, \end{array}$$

where *d*_*i*_ is the Mahalanobis distance of the *i*th observation as defined in (1), and the Huber-type weights are:

$$\begin{array}{l} w_{1}(d)=\{\begin{array}{ll} 1 & \text{if}\;d\leq u\\ u/d & \text{if}\;d>u \end{array},\\ w_{2}(d)={\left[w_{1}(d)\right]}^{2}/\tau , \end{array}$$

and τ is a constant to ensure that ${\hat{\sum }}_{\text{TSR}}$ is unbiased for **Σ**. ${\hat{\mu }}_{\text{TSR}}$ and ${\hat{\sum }}_{\text{TSR}}$ can then be input into common SEM software to obtain robust parameter estimates. However, with TSR, the LRT, fit indices, and standard errors reported in standard software outputs cannot be used; instead, one needs to compute those according to the formulas given in Yuan and Bentler (1998b), which are also available in R using the rsem package (Yuan and Zhang, 2015). An alternative is the direct robust method (Yuan and Zhong, 2008), which uses iteratively reweighted least squares to obtain the Mahanobis distance of each observation based on ${\hat{\text{e}}}_{i}$, downweighs the cases with large Mahanalobis distance, and re-estimates the other model parameters until convergence. For more discussions on TSR and the direct robust method, please consult Yuan and Zhong (2008) and Yuan and Hayashi (2010).

Previous work has shown, mathematically and through empirical examples and simulation studies, that TSR and the direct robust method provided less biased parameter estimates with smaller sampling variability (i.e., greater efficiency) and adjusted LRT relatively insensitive to data contamination. Yuan and Zhong (2013) also demonstrated that TSR and the direct robust method provided fit indices closer to the population value with no outliers or influential observations. However, there are two drawbacks of using Huber-type weights, including (a) the need to select a tuning parameter that determines the proportion of observations being downweighed, and (b) the difficulty in obtaining likelihood-based information criteria for model selection. Therefore, the multivariate-*t* model implemented in Mplus (Muthén and Muthén, 1998–2015), which implicitly also has the effect of downweighing extreme cases but solves the difficulties (a) and (b), will be an attractive alternative to some researchers given its ease of use.

### Multivariate-*t* based SEM

An alternative to handle outliers in regression is to replace the normality assumption on the error terms by a heavy-tailed distribution, where the heavier tails reduce the impact of extreme cases on inferences of the center and variability of the data. One common choice of heavy-tailed distributions is the Student's *t* distribution (Zellner, 1976), with a degree of freedom (*df*) parameter controlling the tail density; a smaller *df* put more weight on the tails, whereas a *df* > 30 effectively makes the *t* distribution closely match the normal distribution. Such a class of models has long been discussed in regression (Gelman and Hill, 2006) and in multilevel modeling (Pinheiro et al., 2001).

In SEM, estimation involving the multivariate *t* distribution is not new. Indeed, as stated in Yuan and Bentler (1998b), by using a specific weighting scheme of the observations in a way analogous to the Huber-type weights, one can obtain robust estimates of mean vector and covariance matrix for the observed variables as the maximum likelihood estimates based on a multivariate *t*-distribution. Yuan and Bentler (1998b) and Yuan et al. (2004) showed that using robust covariances based on the multivariate *t* distribution also performed well in terms of providing less biased parameter estimates and more robust LRT results. However, the procedure discussed in Yuan and Bentler (1998b) required pre-defined degrees of freedom parameter and had not been implemented in statistical software.

Recently, a multivariate-*t* model for SEM has been incorporated into the software Mplus, together with the skew-normal and skew-*t* family (Asparouhov and Muthén, 2015). The documented usage of such models is for mixture modeling with skewed and heavy-tailed compositions to avoid spurious latent classes, and to our knowledge there has been no discussion on using the *t*-based model for robust SEM in the presence of data contamination. The multivariate *t* distribution, *t*_*p*_(**μ**, **Σ**, *df*), is a *p*-variate generalization of the Student's *t* distribution with a single *df* parameter, a location vector μ, and a scale matrix **Σ**. The mean of the distribution is **μ** and the covariance matrix is [*df*/(*df* − 2)]**Σ** for *df* > 2. Therefore, when *df* is small, estimates of parameters in **Σ** in the *t*-based model has a different interpretation than those in the normal-based model.

Continuing from the model defined in Equations (2)–(4), with *t*-based SEM one simply replaces the distributional assumption in (4) by:

(5) $$(e,\xi )\sim t_{p+q}\left(0,\left[\begin{array}{cc} \Theta & 0\\ 0 & \Psi \end{array}\right],df\right).$$

This is equivalent to the model with the conditional distribution **Y**|**X** ~ *t*_*p*_(**μ**, **Σ**, *df*), where

(6) $$\mu =\nu +\Lambda {\left(I-B\right)}^{-1}(\alpha +\Gamma X),$$

(7) $$\Sigma =\Lambda {\left(I-B\right)}^{-1}\Psi {\left[\Lambda {\left(I-B\right)}^{-1}\right]}^{⊤}\Lambda^{⊤}.$$

As the model likelihood can be specified, maximum likelihood can be used to estimate all model parameters as well as *df*, thus avoiding the need to choose a tuning parameter as in using Huber-type weights. This also allows the computation of information criteria such as AIC (Akaike Information Criteria), BIC (Bayesian Information Criteria), and SABIC (sample-size adjusted BIC in Mplus).

Although previous studies have shown that the use of robust covariance matrix based on weights corresponding to a multivariate *t* distribution provided good parameter estimates and LRT statistics similar to those obtained without outliers under ML-Normal (Yuan and Bentler, 1998b), to our knowledge no analytic and simulation studies have evaluated the performance of LRT and fit indices obtained under the multivariate-*t*-based SEM as implemented in Mplus (i.e., ML-*t*), with *df* being estimated instead of specified by users. Given that parameter estimates are not trustworthy when the model fit is sup-optimal, accurate assessment of model fit for an SEM model is of paramount importance. Therefore, this study is an important first step in examining SEM models with ML-*t* as a robust option in handling outliers and influential observations.

It should be clarified that our discussion is limited to robust models that are insensitive to the influence of data contamination, which is different from SEM methods that are robust to non-normality, such as the corrections in LRT and standard errors proposed by Satorra and Bentler (1994). Although there are some commonalities between the two topics, the former focuses on downweighing extreme observations to obtain estimates and inferences when the normality assumption still holds approximately for the majority of the data, and robust SEM methods for non-normality focuses on obtaining inferences when the normality assumption is violated in general. Whereas the latter has received much attention in SEM literature (e.g., Bentler, 1983; Browne, 1984), they generally require estimation of some higher moments of the sample data, which would be highly unstable in the presence of data contamination. Indeed, as would be mentioned in the discussion, we found the Satorra-Bentler correction performed sub-optimally in the presence of data contamination.

## Real data demonstration

We now briefly demonstrate the use of SEM with ML-*t* using the classic Holzinger and Swineford (1939) data set and a modified version where five observations were changed to have strong influence to model fit. The nine variables are cognitive test scores for 145 students. Using ML-Normal with the original data set, a 3-factor CFA model with a cross-loading of item 9 on factor 1 fit the data well, with χ^2^(*df*=23, *N*=145)=28.29, *p*=0.205, *RMSEA*=0.040, *CFI*=0.989, *SRMR*=0.040. One can also use the DIST = TDISTRIBUTION option in Mplus to fit the same model with *t*-likelihood, and add the OUTPUT: H1MODEL option to obtain χ^2^ statistic and fit indices. Using the same data, one obtains χ^2^(*df*=23, *N*=145)=25.29, *p*=0.335, *RMSEA*=0.026, *CFI*=0.994, which were close to the values with ML-Normal. SRMR is not yet obtainable as it does not directly depend on χ^2^. The estimated *df* using maximum likelihood is 24.5, with a 95% confidence interval (CI) of [15.9, 34.1]. Using TSR as implemented in the R package rsem with the same CFA model and data, we have χ^2^(*df*=23, *N*=145)=26.82, *p*=0.264, *RMSEA*=0.034, *CFI*=0.992, *SRMR*=0.038. AIC, BIC, and SABIC all preferred ML-*t* over ML-Normal with differences of 8.5, 5.5, and 8.7 respectively, indicating some evidence for slightly heavier tails in the sample distributions even without modifications.^1^ However, the fit information and parameter estimates (as shown in Table 1) under all three methods were similar, so the choice is trivial and one can be more confident that data contamination should not be an issue.

**Table 1:** Parameter estimates and standard errors from the real data demonstration.

	**ML-Normal**		**ML-*****t***		**TSR**	
**Parameters**	**Original**	**Modified**	**Original**	**Modified**	**Original**	**Modified**
λ_11_	1.00 (—)	1.00 (—)	1.00 (—)	1.00 (—)	1.00 (—)	1.00 (—)
λ_21_	0.66 (0.14)	1.14 (0.06)	0.63 (0.14)	0.73 (0.13)	0.62 (0.13)	0.77 (0.14)
λ_31_	0.84 (0.15)	0.43 (0.04)	0.82 (0.15)	0.74 (0.12)	0.78 (0.13)	0.73 (0.12)
λ_42_	1.00 (—)	1.00 (—)	1.00 (—)	1.00 (—)	1.00 (—)	1.00 (—)
λ_52_	0.99 (0.09)	1.47 (0.06)	1.02 (0.09)	1.09 (0.10)	1.04 (0.09)	1.07 (0.09)
λ_62_	0.96 (0.08)	1.14 (0.05)	0.97 (0.09)	0.95 (0.08)	0.96 (0.09)	0.93 (0.08)
λ_73_	1.00 (—)	1.00 (—)	1.00 (—)	1.00 (—)	1.00 (—)	1.00 (—)
λ_83_	1.27 (0.23)	1.49 (0.06)	1.27 (0.24)	1.37 (0.20)	1.18 (0.19)	1.29 (0.16)
λ_91_	0.56 (0.12)	1.18 (0.20)	0.55 (0.12)	0.56 (0.12)	0.51 (0.11)	0.52 (0.14)
λ_93_	0.64 (0.14)	0.49 (0.21)	0.63 (0.14)	0.66 (0.13)	0.65 (0.13)	0.69 (0.14)
ψ_11_	0.67 (0.16)	3.95 (0.55)	0.64 (0.16)	0.72 (0.16)	0.70 (0.16)	0.81 (0.21)
ψ_22_	0.94 (0.15)	3.11 (0.42)	0.85 (0.15)	0.85 (0.15)	0.87 (0.14)	0.97 (0.16)
ψ_33_	0.50 (0.13)	3.33 (0.47)	0.46 (0.13)	0.52 (0.13)	0.50 (0.13)	0.62 (0.14)

*ML, maximum likelihood; TSR, Two-stage robust methods with Huber-type weights downweighing 10% of observations; λ, factor loading; ψ, factor variance. Standard errors were shown in parentheses*.

We then use a modified data set described in Yuan and Zhong (2013, p. 131, Data Set D4) containing five bad influential observations (i.e., 3%). The Mahanalobis distances of the five modified observations were between 4.22 and 128.26, compared to 1.41 to 24.84 for the other observations. ML-Normal gave χ^2^(*df*=23, *N*=145)=57.80, *p* < 0.001, *RMSEA*=0.095, *CFI*=0.984, *SRMR*=0.020. Although both χ^2^ and RMSEA indicated worse model fit, the impact on CFI was small and SRMR actually indicated better model fit. Therefore, with the presence of bad influential observations, ML-Normal gave ambiguous fit information. ML-*t*, on the other hand, gave χ^2^(*df*=23, *N*=145)=24.81, *p*=0.360, *RMSEA*=0.023, *CFI*=0.996; TSR gave χ^2^(*df*=23, *N*=145)=30.55, *p*=0.134, *RMSEA*=0.048, *CFI*=0.987, *SRMR*=0.037. Thus, the fit information using ML-*t* or TSR was comparable with or without the bad influential cases, and AIC, BIC, and SABIC all strongly favored ML-*t* over ML-Normal with differences in values of more than 300. The parameter estimates were shown in Table 1, which shows that whereas estimates were strongly affected by the influential observations using ML-Normal, they were robust with ML-*t* and TSR.

## Simulation study

We conducted a Monte Carlo simulation study to compare the performance of fit indices (χ^2^, RMSEA, and CFI) under ML-Normal, ML-*t*, and TSR, as well as model comparisons between ML-Normal and ML-*t* using AIC, BIC, and SABIC. Note that SRMR is not available in Mplus 7.4 with ML-*t*. A 2 (type of data contamination) × 4 (proportion of outliers/influential observations) × 2 (model misspecification) × 3 (sample size) design was used, as described later. For all simulation conditions, data were generated first from a 3-factor 9-indicator model similar to the previous real data demonstration, which was also used in Zhong and Yuan (2011), with a cross-loading of item 9 on Factor 3. Specifically,

$$\begin{array}{l} \Lambda ={\left[\begin{array}{ccccccccc} 1.0 & 0.9 & 1.1 & 0 & 0 & 0 & 0 & 0 & 0.5\\ 0 & 0 & 0 & 1.0 & 0.7 & 0.55 & 0 & 0 & 0\\ 0 & 0 & 0 & 0 & 0 & 0 & 1 & 1.3 & 1.25 \end{array}\right]}^{⊤},\\ \Psi =\left[\begin{array}{ccc} 1 & 0.25 & 0.25\\ 0.25 & 1 & 0.25\\ 0.25 & 0.25 & 1 \end{array}\right], \end{array}$$

and **Θ** = **I** is a 9 × 9 identity matrix. All factor means and intercepts were set to zero for simplification. For each simulation condition, we ran 2,000 replications.

**Figure 1:**
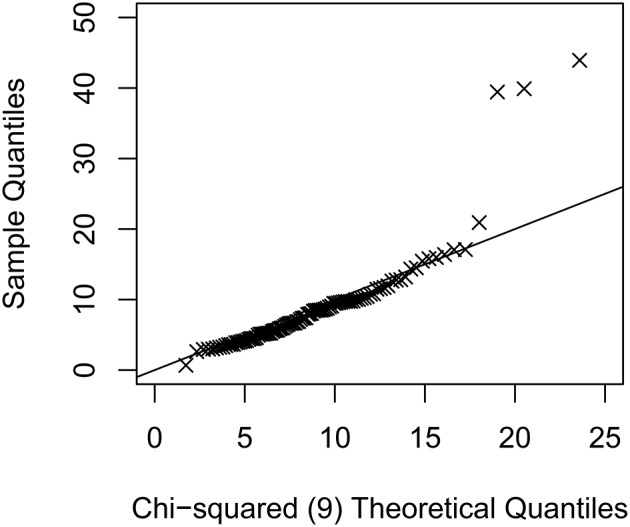
An example quantile-quantile plot of the sample Mahanalobis distance against the theoretical values of a chi-squared distribution with 9 degrees of freedom for the condition with *N* = 100 and 5 outliers. Multivariate kurtosis = 9.66 for this simulated data set.

Factor scores, **ξ** ~ *N*(**0**, **Ψ**), and multivariate normal data, denoted as **y**^(1)^, were generated according to the above model using R 3.3.1 (R Core Team, 2016), and outliers or bad influential observations were introduced using methods described in Zhong and Yuan (2011). Let ε = 0, 0.05, 0.075, or 0.10 be the proportion of cases that are either outliers or bad influential observations. For conditions with outliers, ε*N* observations were modified as ${\text{y}}_{i}^{\text{mod}}={\text{y}}_{i}^{\left(1\right)}+h_{i}\Lambda^{o}\xi_{i}$, where *h*_*i*_ ~ exp(*z*_*i*_) and *z*_*i*_s were randomly generated from independent *N*(0, 1) distributions and varied across replications; **Λ**^*o*^ is the modified loading matrix such that **Λ**^⊤^**Λ**^*o*^ = **0** as discussed in Yuan and Zhong (2013). For conditions with bad influential observations, ε*N* observations were modified as ${\text{y}}_{i}^{\text{mod}}=h_{i}{\text{y}}_{i}^{\left(1\right)}$. The sample size (*N*) was either 100, 200, or 500, as most studies using SEM had *N* ≥ 100 (Jackson et al., 2009), and SEM was generally not recommended with a small sample size (Kline, 2011). The Mahalanobis distances for the outliers or influential observations varied across replications, and for each replication the maximum *M*-distance was 15.8–76.0, 19.7–149.5, 14.5–350.1 for *N* = 100, 200, and 500 with outliers (see Figure 1 for an example), and 29.1–92.2, 30.3–190.4, 26.7–472.5 for *N* = 100, 200, and 500 with bad influential observations. For each generated data set, we either fit a correctly specified model or a misspecified model with no cross-loading (with population RMSEA = 0.061, population CFI = 0.96), and for each model we used either ML-Normal or ML-*t* and obtain fit indices in Mplus, and used rsem to obtain fit indices with TSR (10% observations downweighed).

### Evaluation criteria

For each condition, we evaluated the rejection rates of LRT at *p* < 0.05 using the three methods, with rates close to 5% being optimal for conditions with a correctly specified model, and rates close to the empirical power with no data contamination best for conditions with a misspecified model. For RMSEA and CFI we graphically examine the distributions of the sample fit values, with preference given to methods providing sample fit values close to population fit values and with small variabilities across replications. Finally, we examined the empirical probability of information criteria selecting ML-*t* over ML-Normal (i.e., having smaller values for ML-*t*).

### Simulation results

#### Convergence

Convergence rates were above 90.5% for ML-Normal and above 93.3% for ML-*t* for conditions with correctly specified model, and were above 89.7% and above 87.8% for conditions with misspecified model. Convergence was better for conditions with *N* ≥ 200 (> 98.3% for ML-*t* and > 92.5% for ML-Normal); it was worst for ML-Normal with 10% bad influential observations (90.5% for *N* = 100 and 92.8% for *N* = 200 for correctly specified model and 89.7% for *N* = 100 and 92.5% for misspecified model), and for ML-*t* with small sample size (93.3–95.2% for correctly specified model and 87.8–91.0% for misspecified model when *N* = 100). Convergence was generally better for ML-*t* than for ML-Normal in conditions with outliers or bad influential observations when *N* ≥ 200. Convergence rates were at least 99.2% with TSR.

#### Fit indices

**Figure 2:**
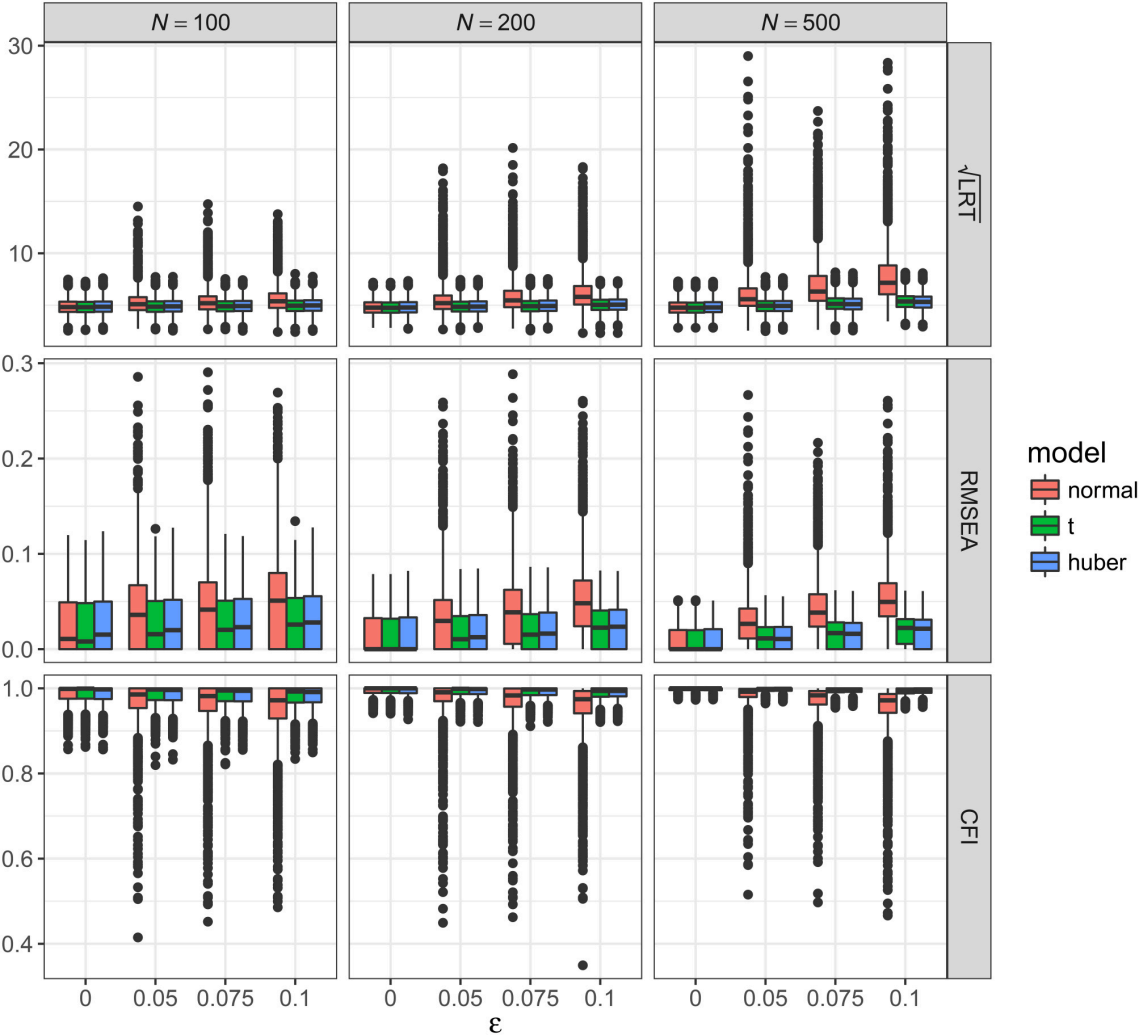
Values of fit indices across simulation conditions with a correctly specified model and the presence of outliers. LRT, likelihood ratio test; ε, proportion of outliers. Square root was taken on the LRT values to reduce its skewness for better graphical presentations.

**Figure 3:**
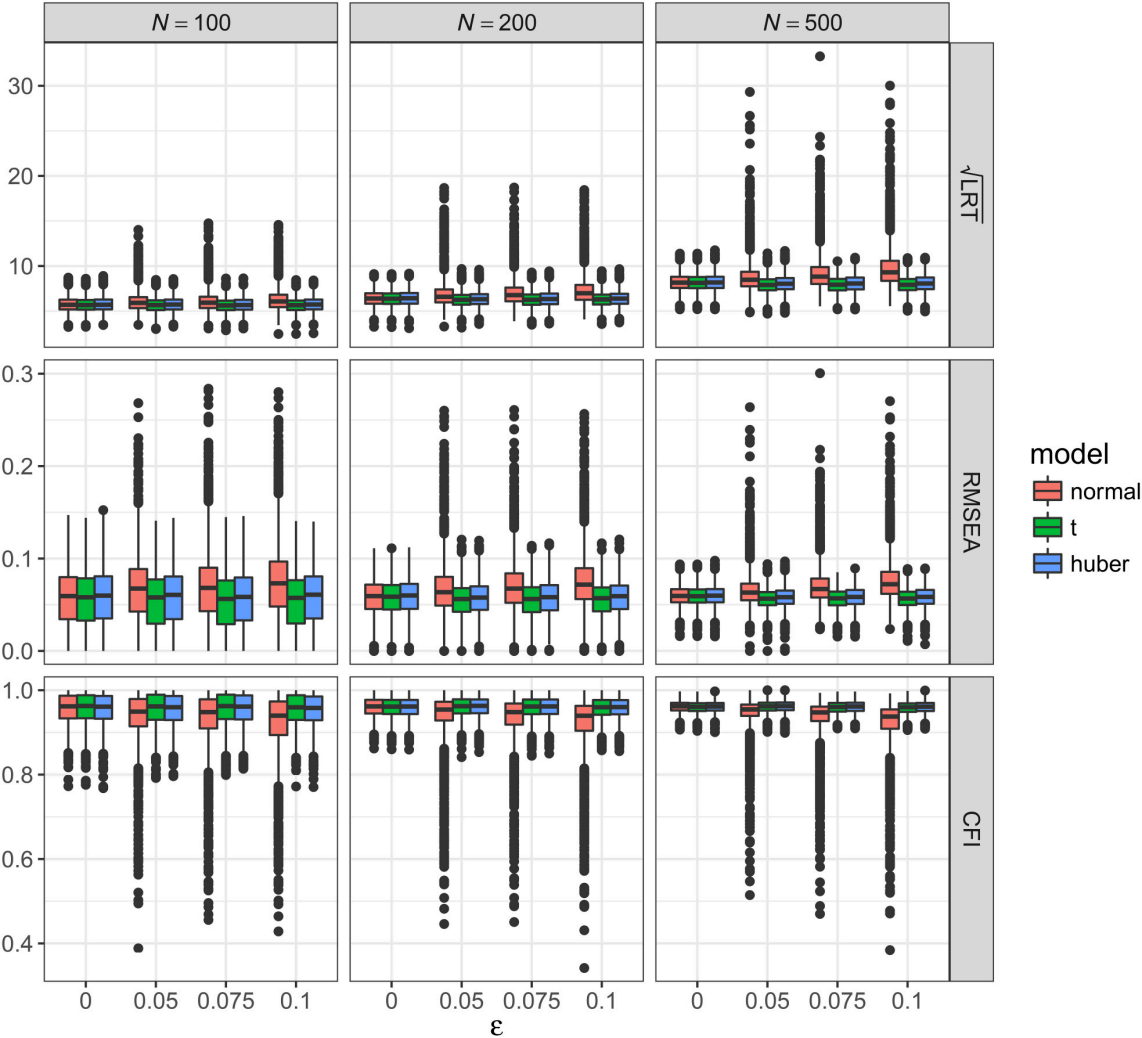
Values of fit indices across simulation conditions with a misspecified model and the presence of outliers. LRT, likelihood ratio test; ε, proportion of outliers. Square root was taken on the LRT values to reduce its skewness for better graphical presentations.

**Figure 4:**
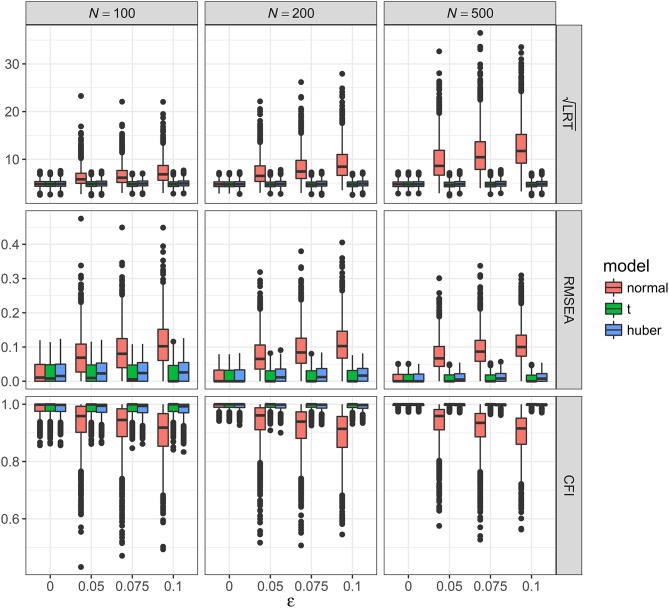
Values of fit indices across simulation conditions with a correctly specified model and the presence of bad influential observations. LRT, likelihood ratio test; ε, proportion of bad influential observations. Square root was taken on the LRT values to reduce its skewness for better graphical presentations.

**Figure 5:**
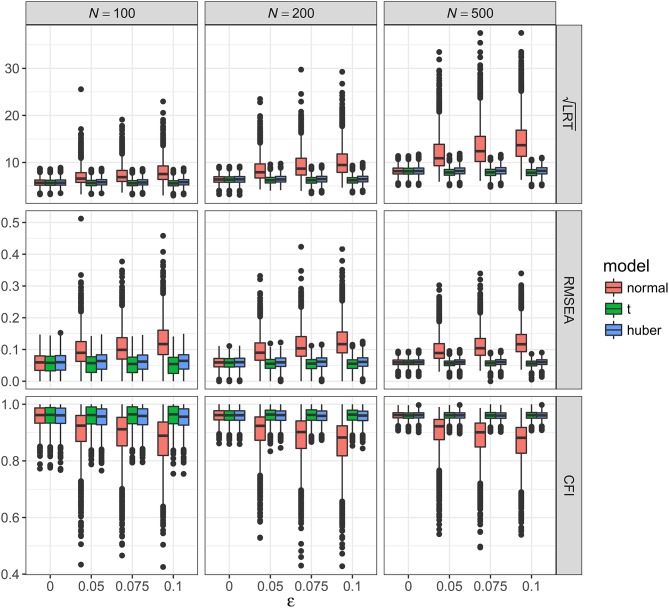
Values of fit indices across simulation conditions with a misspecified model and the presence of bad influential observations. LRT, likelihood ratio test; ε, proportion of bad influential observations. Square root was taken on the LRT values to reduce its skewness for better graphical presentations.

As shown in Figures 2–5, the results of ML-*t* and TSR were almost identical, so in the following sections we mainly prsented results for ML-Normal and ML-*t*.

##### Outliers with correctly specified model

Figure 2 showed the boxplots of sample values of LRT, RMSEA, and CFI for conditions with a correctly specified model and the presence of outliers. With ML-Normal, LRT, RMSEA, and CFI became substantially worse and more variable with increasing proportion of outliers. When *N* = 100, median LRT increased slightly from 23.26 (adjusted median absolute deviation, *SD* = 7.28) with no outliers to 28.96 (*SD* = 19.63) with 10% outliers; when *N* = 500, median LRT increased dramatically from 22.73 (*SD* = 6.82) with no outliers to 51.27 (*SD* = 71.74) with 10% outliers. Empirical Type I error rates of LRT were inflated from 5.4 to 7.3% with no outliers to 31.0–77.7% with 10% outliers (see Table 2). For RMSEA and CFI, the impact of *N* was smaller: when *N* = 500, median RMSEA increased from 0.000 (*SD* = 0.012) to 0.050 (*SD* = 0.035); median CFI decreased from 1.00 (*SD* = 0.004) to 0.972 (*SD* = 0.066). Simiar trends were observed for *N* = 100 and *N* = 200.

**Table 2:** Rejection rates of the likelihood ratio test across conditions.

				**Outliers**			**Bad Influential Observations**		
**Model**	***N***	**Estimation**	**ε = 0**	**ε = 0.05**	**ε = 0.075**	**ε = 0.10**	**ε = 0.05**	**ε = 0.075**	**ε = 0.10**
Correct	100	ML-Normal	0.07	0.20	0.23	0.31	0.47	0.55	0.68
		ML-*t*	0.07	0.09	0.08	0.10	0.07	0.07	0.07
		TSR	0.08	0.09	0.09	0.11	0.09	0.09	0.11
	200	ML-Normal	0.06	0.25	0.37	0.46	0.62	0.76	0.84
		ML-*t*	0.06	0.08	0.11	0.12	0.05	0.05	0.05
		TSR	0.07	0.08	0.11	0.12	0.08	0.08	0.09
	500	ML-Normal	0.05	0.40	0.61	0.78	0.85	0.94	0.98
		ML-*t*	0.05	0.09	0.17	0.23	0.05	0.06	0.05
		TSR	0.06	0.09	0.16	0.22	0.08	0.09	0.09
Misspecified	100	ML-Normal	0.35	0.44	0.46	0.52	0.67	0.74	0.82
		ML-*t*	0.33	0.32	0.30	0.32	0.31	0.30	0.29
		TSR	0.34	0.35	0.33	0.35	0.39	0.37	0.40
	200	ML-Normal	0.66	0.72	0.77	0.82	0.90	0.94	0.97
		ML-*t*	0.66	0.60	0.60	0.63	0.59	0.59	0.58
		TSR	0.67	0.64	0.64	0.65	0.67	0.69	0.69
	500	ML-Normal	0.99	0.99	1.00	1.00	1.00	1.00	1.00
		ML-*t*	0.99	0.98	0.98	0.98	0.98	0.97	0.98
		TSR	0.99	0.98	0.99	0.99	0.99	0.99	0.99

*Note. ε, Proportion of outliers and bad influential observations; ML, maximum likelihood estimation; TSR, Two-stage robust methods with Huber-type weights downweighing 10% of observations*.

With ML-*t*, LRT, RMSEA, and CFI were relatively stable with increasing proportion of outliers. When *N* = 100, LRT were similar with no outliers, median = 23.15 (*SD* = 7.26), and with 10% outliers, 24.53 (*SD* = 7.51); when *N* = 500, median LRT increased slightly from 22.56 (*SD* = 6.81) with no outliers to 28.71 (*SD* = 8.63) with 10% outliers. Empirical Type I error rates were inflated from 5.2 to 7.2% with no outliers to 10.5–22.7% with 10% outliers. For RMSEA and CFI, when *N* = 500, median RMSEA increased from 0.000 (*SD* = 0.012) to 0.022 (*SD* = 0.015); median CFI decreased from 1.00 (*SD* = 0.004) to 0.994 (*SD* = 0.008). Simiar trends were observed for *N* = 100 and *N* = 200.

##### Outliers with misspecified model

Figure 3 showed the boxplots of sample values of LRT, RMSEA, and CFI for conditions with a misspecified model and the presence of outliers. In general, the patterns were similar to those observed with a correctly specified model, except that, predictably, the fit was worse on all conditions. With ML-Normal, when *N* = 100, median LRT increased slightly from 32.45 (*SD* = 9.37) with no outliers to 36.84 (*SD* = 20.88) with 10% outliers; when *N* = 500, median LRT increased from 66.52 (*SD* = 15.33) with no outliers to 86.52 (*SD* = 71.38) with 10% outliers. Empirical power of LRT was inflated from 34.6 to 99.1% with no outliers to 51.5–99.8% with 10% outliers (see Table 2). For RMSEA and CFI, when *N* = 500, median RMSEA increased from 0.060 (*SD* = 0.011) to 0.072 (*SD* = 0.026); median CFI decreased from 0.962 (*SD* = 0.013) to 0.938 (*SD* = 0.064). Simiar trends were observed for *N* = 100 and *N* = 200.

With ML-*t*, LRT, RMSEA, and CFI were relatively stable with increasing proportion of outliers and remained close to the population values without data contamination, with medians and *SD*s stayed virtually the same regardless of ε. Empirical power was 32.7–99.0% with no outliers and 31.8–98.3% with 10% outliers.

##### Bad influential observations with correctly specified model

Figure 4 showed the boxplots of sample values of LRT, RMSEA, and CFI for conditions with a correctly specified model and the presence of bad influential observations. Given the nature of such observations, their presence made a bigger impact on LRT, RMSEA, and CFI than outliers did. When *N* = 100, median LRT increased slightly from 23.26 (*SD* = 7.28) with no influential observations to 47.02 (*SD* = 41.39) with 10% influential observations; when *N* = 500, median LRT increased dramatically from 22.73 (*SD* = 6.82) with no influential observations to 137.98 (*SD* = 139.53) with 10% influential observations. Empirical Type I error rates were inflated from 5.4 to 7.3% with no influential observations to 67.8–97.7% with 10% influential observations (see Table 2). When *N* = 500, median RMSEA increased from 0.000 (*SD* = 0.012) to 0.100 (*SD* = 0.047), and median CFI decreased from 1.00 (*SD* = 0.004) to 0.915 (*SD* = 0.071). Simiar trends were observed for *N* = 100 and *N* = 200.

With ML-*t*, LRT, RMSEA, and CFI were relatively stable with increasing proportion of influential observations, with medians and *SD*s stayed virtually the same regardless of ε. Empirical Type I error rates were 4.4–7.1% with no influential observations and 4.2–7.2% with 10% influential observations.

##### Bad influential observations with misspecified model

Figure 5 showed the boxplots of sample values of LRT, RMSEA, and CFI for conditions with a misspecified model and the presence of bad influential observations. In general, the patterns were similar to those observed with a correctly specified model, except that, predictably, the fit was worse on all conditions. With ML-Normal, when *N* = 100, median LRT increased from 32.45 (*SD* = 9.37) with no outliers to 56.60 (*SD* = 43.27) with 10% influential observations; when *N* = 500, median LRT increased from 66.52 (*SD* = 15.33) with no outliers to 186.28 (*SD* = 153.70) with 10% influential observations. Empirical power was inflated from 34.6 to 99.1% with no outliers to 82.3–100.0% with 10% outliers (see Table 2). For RMSEA and CFI, when *N* = 500, median RMSEA increased from 0.060 (*SD* = 0.011) to 0.116 (*SD* = 0.043); median CFI decreased from 0.962 (*SD* = 0.013) to 0.882 (*SD* = 0.073). Simiar trends were observed for *N* = 100 and *N* = 200.

With ML-*t*, LRT, RMSEA, and CFI were relatively stable with increasing proportion of influential observations and remained close to the population values without data contamination, with medians and *SD*s stayed virtually the same regardless of ε. Empirical power was 32.7–99.0% with no outliers and 29.4–97.6% with 10% outliers.

#### Information criteria

**Figure 6:**
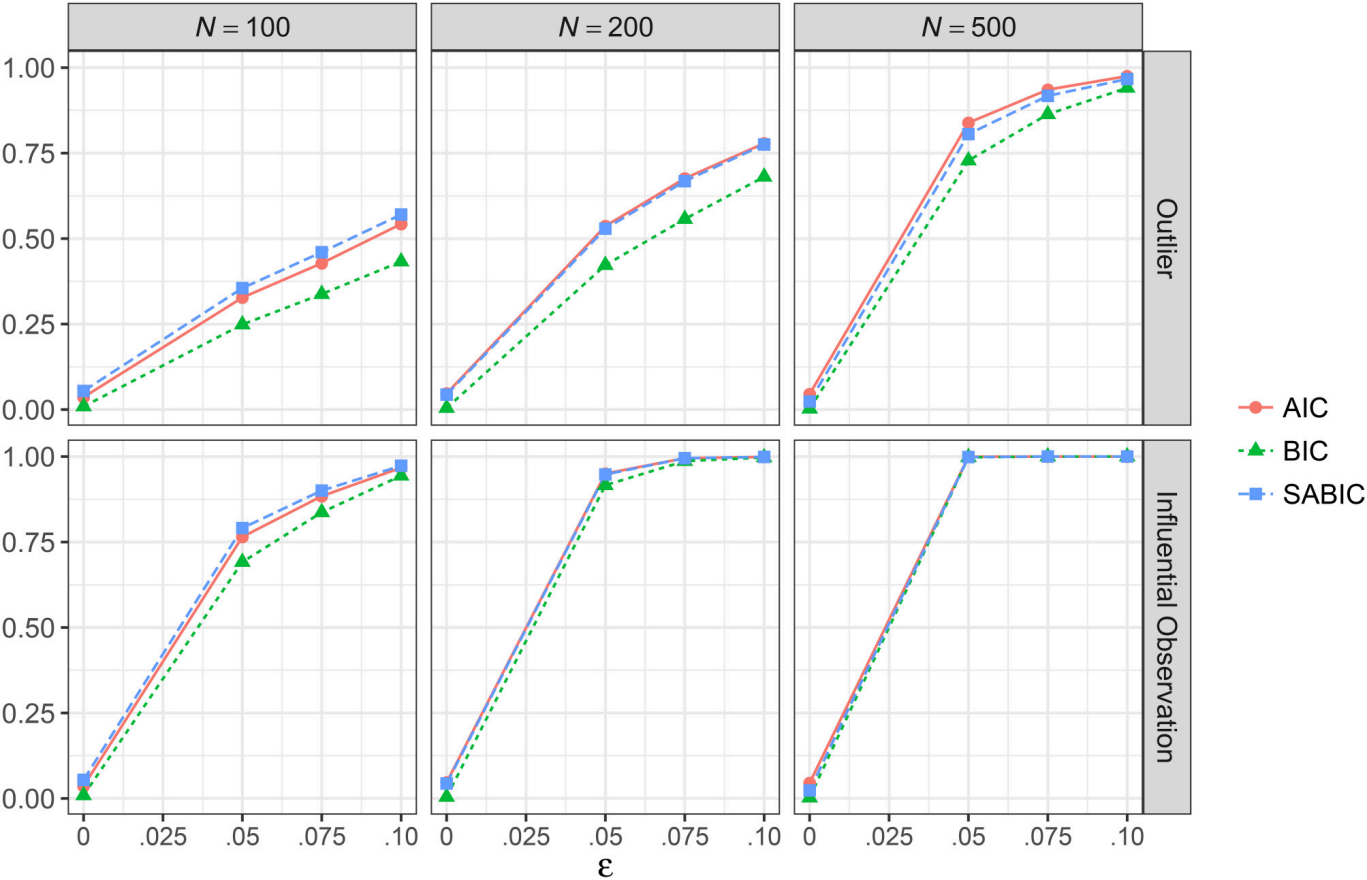
Proportion of replications where the multivariate *t* model has smaller information criteria than the multivariate normal model across simulation conditions with a correctly specified model. AIC, Akaike information criteria; BIC, Bayesian information criteria; SABIC, sample-size adjusted information criteria; ε, proportion of outliers.

Figure 6 showed the proportion of replications where AIC, BIC, and SABIC favored ML-*t* over ML-Normal for conditions with a correctly specified model, and the results were essentially identical for conditions with a misspecified model. Under a correctly specified model, with no outliers or influential observations, in only 3.6–4.7% of the replications ML-*t* was preferred over ML-Normal by AIC, 0.3–0.9% by BIC, and 2.4–5.4% by SABIC; with 10% outliers, ML-*t* was preferred more often with increasing proportion of outliers and with larger *N*, with AIC, BIC, and SABIC favoring ML-*t* in 97.5, 94.1, and 96.7% of the replications when *N* = 500. Similarly, with influential observations, AIC, BIC, and SABIC preferred ML-*t* in 76.5, 69.2, and 79.1% of the replications when ε = 0.05 and *N* = 100 and well above 90% for all conditions with either ε = 0.10 or *N* ≥ 200.

## Discussion

Although the impact of and ways to handle outliers and influential observations have received much attention in regression literature, relatively less discussions on those issues were found in the context of SEM. As pointed out in Yuan and Zhong (2013), unlike general statistics software where diagnostic tools for outliers and influential observations are common, such tools are rarely accessible for SEM software, partly because of the complexity of SEM modeling. Whereas robust SEM using Huber-type weights has been developed and shown to perform well, and the rsem package is freely available in R, many researchers are more familiar with other commonly used SEM software packages such as Mplus, and so it is important to have comparable tools for handling outliers and influential observations in other software. With the *t*-based model recently added to Mplus, this study brings attention to this easy-to-use strategy to clarify whether suboptimal model fit is due to global misfit or just a small proportion of extreme cases.

Our simulation results showed that outliers and influential observations could hurt model convergence and dramatically make model fit appear worse for both correctly specified and misspecified SEM models with the usual ML estimation assuming normality. For example, with 25 outliers in a sample of 500 observations, the empirical Type I error rate for LRT was inflated to 0.40 from the nominal level of 0.05, and it was inflated to 0.85 with 25 bad influential observations. Both RMSEA and CFI were more likely to indicate worse model fit in the presence of outliers and influential observations, as predicted in Yuan and Zhong (2013). As it was not common that applied researchers check for outliers and influential observations when conducting SEM (Aguinis et al., 2013), such extreme values may make researchers reject models with adequate fit or consider alternative models that improve overall model fit mainly because of those few observations.

On the other hand, the multivariate-*t* model as well as the two-stage robust method were more robust to data contamination, producing fit indices that were closer to what could have been obtained without those extreme values. First, when sample size was small (*N* = 100) ML-*t* may have some convergence problems in 5–7% of the replications with no model misspecifications and in 9–12% of the replications with misspecifications; however, with *N* = 200 or above the use of ML-*t* had improved convergence rates over ML-Normal. Second, although to a much less degree, with ML-*t* and TSR, LRT still increased with increasing proportion of outliers, and empirical Type I error rates increased to 0.10 and 0.11 for *N* = 100 and 0.23 and 0.22 for *N* = 500 with 10% of outliers. Although this is certainly not ideal, LRT under ML-*t* or TSR still performs much better than under ML-Normal. Future studies can focus on how to obtain adjusted test statistics for ML-*t*. Note, however, when the model was misspecified, or when the extreme values were bad influential observations, LRT, RMSEA, and CFI were all similar regardless of proportions of data contamination, and the values under ML-*t* were slightly closer to the population values than those under TSR.

Third, information criteria was effective in picking ML-Normal when no outliers or bad influential observations were present in the data, and in picking ML-*t* when extreme values were present, with better accuracy when sample size increased. Under our simulation conditions, we found AIC and SABIC showed higher sensitivity than BIC. Therefore, when researchers are uncertain whether data contamination could be a problem, an effective way in determining whether to use ML-Normal or ML-*t* is to choose one that gives smaller AIC and SABIC.

It is generally recommended to use multivariate-*t*-based SEM and other robust SEM methods, rather than directly deleting outliers and influential observations, as the complexity of SEM makes it more challenging to use general techniques such as Mahalanobis distance and Cook's distance to identify outliers and influential observations (e.g., Flora et al., 2012; Sterba and Pek, 2012). Although these methods provided parameter estimates and fit indices that are insensitive to the influence of outliers, they do not replace the need for careful data screening work. As suggested by Aguinis et al. (2013), one should always identify in the data if there are any extreme cases due to correctible errors, and correct them accordingly. Failure to do so may lead to loss of valuable information. Also, after any such errors are corrected, the use of robust SEM is justified only when the outliers and influential observations are regarded as coming from a different data generating process than the majority of the data, and the goal of inference is to estimate a model that is representative of most of the data. Sometimes outliers and influential observations can be of interest in their own rights, and they can lead to important research findings (Aguinis et al., 2013; O'Connell et al., 2015). Also, a non-trivial proportion of such cases may indicate unmodeled heterogeneity, where the use of mixture models may be more appropriate. Recent methodological work has provided accessible tools to identify outliers and influential observations for SEM (Pek and MacCallum, 2011; Sterba and Pek, 2012), which we recommend to be used in combination with robust SEM methods.

Despite the contributions of the study, there are several limitations that call for future studies. First, as a first step to evaluate the multivariate-*t*-based SEM, we chose to first study the performance of fit indices under such a model. An obvious next step is to make sure that the parameter estimates are sensible with the multivariate-*t*-based SEM, which appeared to be robust based on the real data example and the results in Yuan and Bentler (1998b) using weights corresponding to a multivariate *t* distribution with one degree of freedom. Second, as pointed out in Yuan et al. (2004), the use of multivariate-*t*-based SEM might not be as efficient as the use of Huber-type weights under some conditions, and future studies may compare the performance of various robust methods in simulated and real data. At this stage, we found that the use of the multivariate-*t*-based SEM is accessible to researchers without the need to choose a tuning parameter, allows conventional interpretations of information criteria, and can be easily integrated into more complex SEM models.

Third, it should be emphasized again that, in the current study, we only focused on situations where a small proportion of data is contaminated, whereas the majority of the data still satisfies the normality assumption. Although the resulting data also had skewness and kurtosis deviated from those of a normal distribution, common SEM estimation methods that are robust to non-normality may not work well in the presence of data contamination. Whereas corrections for non-normality such as the Satorra-Bentler procedure relies on sandwich estimator and higher-order moments of the sample data, ML-*t* as implemented in Mplus uses maximum likelihood with the expectation-maximization algorithm to estimate the model parameters, including degrees of freedom. To examine our speculations, we re-analyze the simulated data using the Satorra-Bentler correction procedure (ESTIMATOR=MLM in Mplus), and found the resulting fit indices to still be sensitive to data contamination, although not to the extent as ML-Normal. For example, with *N* = 500, 10% outliers, and a correctly specified model, median *RMSEA* = 0.041 with Satorra-Bentler, commpared to 0.050 for ML-Normal and 0.022 for ML-*t*. Therefore, researchers should distinguish between robustness against data contamination, which can be handled with ML-*t* or Huber-type weights, and robustness against non-normality, which can be alleviated by the Satorra-Bentler correction or weighted least squares estimator.

Fourth, our simulations did not cover sample sizes smaller than 100. We had performed additional simulations with *N* = 50 and found that TSR had very low convergence rates (less than 5%) and ML-*t* had convergence around 71–76%, and information criteria preferred ML-*t* only 20% of the replications in the presence of 10% outliers observations. Therefore, we recommend using ML-*t* or the two-stage robust methods only with a sample size of at least 100. Finally, in this study we only evaluated fit indices with a factor model, and consider only misspecifications in the form of missing one cross-loading. As the impact of outliers and influential observations on fit indices may vary depending on types of SEM models, model complexity, and parameter values, future studies can expand on the simulation conditions to provide more complete information on this underresearched area.

## Author contributions

ML designed the simulation and drafted the manuscript. JZ helped conducting the simulation, provided recommendations on the draft, and proofread.

### Conflict of interest statement

The authors declare that the research was conducted in the absence of any commercial or financial relationships that could be construed as a potential conflict of interest.

## Footnotes

^1^It is still an open question how information criteria should be defined when using TSR, and whether the values obtained can be compared with information criteria obtained using maximum likelihood. Therefore, we did not report information criteria with TSR.
